# Usability of Health Care Price Transparency Data in the United States: Mixed Methods Study

**DOI:** 10.2196/50629

**Published:** 2024-03-29

**Authors:** Negar Maleki, Balaji Padmanabhan, Kaushik Dutta

**Affiliations:** 1 School of Information Systems and Management Muma College of Business University of South Florida Tampa, FL United States; 2 Decision, Operations & Information Technologies Department Robert H. Smith School of Business University of Maryland College Park, MD United States

**Keywords:** price transparency, user experiments, schema analysis, health care, patients, algorithms

## Abstract

**Background:**

Increasing health care expenditure in the United States has put policy makers under enormous pressure to find ways to curtail costs. Starting January 1, 2021, hospitals operating in the United States were mandated to publish transparent, accessible pricing information online about the items and services in a consumer-friendly format within comprehensive machine-readable files on their websites.

**Objective:**

The aims of this study are to analyze the available files on hospitals’ websites, answering the question—is price transparency (PT) information as provided usable for patients or for machines?—and to provide a solution.

**Methods:**

We analyzed 39 main hospitals in Florida that have published machine-readable files on their website, including commercial carriers. We created an Excel (Microsoft) file that included those 39 hospitals along with the 4 most popular services—Current Procedural Terminology (CPT) 45380, 29827, and 70553 and Diagnosis-Related Group (DRG) 807—for the 4 most popular commercial carriers (Health Maintenance Organization [HMO] or Preferred Provider Organization [PPO] plans)—Aetna, Florida Blue, Cigna, and UnitedHealthcare. We conducted an A/B test using 67 MTurkers (randomly selected from US residents), investigating the level of awareness about PT legislation and the usability of available files. We also suggested format standardization, such as master field names using schema integration, to make machine-readable files consistent and usable for machines.

**Results:**

The poor usability and inconsistent formats of the current PT information yielded no evidence of its usefulness for patients or its quality for machines. This indicates that the information does not meet the requirements for being consumer-friendly or machine readable as mandated by legislation. Based on the responses to the first part of the experiment (PT awareness), it was evident that participants need to be made aware of the PT legislation. However, they believe it is important to know the service price before receiving it. Based on the responses to the second part of the experiment (human usability of PT information), the average number of correct responses was not equal between the 2 groups, that is, the treatment group (mean 1.23, SD 1.30) found more correct answers than the control group (mean 2.76, SD 0.58; *t*_65_=6.46; *P*<.001; *d*=1.52).

**Conclusions:**

Consistent machine-readable files across all health systems facilitate the development of tools for estimating customer out-of-pocket costs, aligning with the PT rule’s main objective—providing patients with valuable information and reducing health care expenditures.

## Introduction

### Overview

From 1970 to 2020, on a per capita basis, health care expenditures in the United States have increased sharply from US $353 per person to US $12,531 per person. In constant 2020 dollars, the increase was from US $1875 in 1970 to US $12,531 in 2020 [1]. The significant rise in health care expenses has put policy makers under enormous pressure to find ways to contain these expenditures. Price transparency (PT) in health care is 1 generally proposed strategy for addressing these problems [2] and has been debated for years [3]. Some economists believe that PT in health care will cut health care prices in the same way it has in other industries, while others argue that owing to the specific characteristics of the health care market, PT would not ameliorate rising health care costs. Price elasticity also does not typically apply in health care, since, if a problem gets severe, people will typically seek treatment regardless of cost, with the drawback that individuals learn of their health care costs after receiving treatment [4]. Complex billing processes, hidden insurer-provider contracts, the sheer quantity of third-party payers, and substantial quality differences in health care delivery are other unique aspects of health care that complicate the situation considerably.

The Centers for Medicare & Medicaid Services (CMS) mandated hospitals to post negotiated rates, including payer-specific negotiated costs, for 300 “shoppable services” beginning in January 2021. The list must include 70 CMS-specified services and an additional 230 services each hospital considers relevant to its patient population. Hospitals must include each third-party payer and their payer-specific fee when negotiating multiple rates for the same care. The data must be displayed simply, easily accessible (without requiring personal information from the patient), and saved in a machine-readable manner [5]. These efforts aim to facilitate informed patient decision-making, reduce out-of-pocket spending, and decrease health care expenditures. Former Secretary of Health and Human Services, Alex Azar, expressed a vision of hospital PT when declaring the new legislation “a patient-centered system that puts you in control and provides the affordability you need, the options and control you want, and the quality you deserve. Providing patients with clear, accessible information about the price of their care is a vital piece of delivering on that vision” [6].

Despite the legislation, it is not clear if people are actually engaging in using PT tools. For example, in 2007, New Hampshire’s HealthCost website was established, providing the negotiated price and out-of-pocket costs for 42 commonly used services by asking whether the patient is insured or their insurer and the zip code to post out-of-pocket costs in descending order. Mehrotra et al [7] examined this website over 3 years to understand how often and why these tools have mainly been used. Their analysis suggested that despite the growing interest in PT, approximately 1% of the state’s population used this tool. Low PT tool usage was also seen in other studies [8-10], suggesting that 3% to 12% of individuals who were offered the tool used it during the study period, and in all studies, the duration was at least 12 months. Thus, offering PT tools does not in itself lead to decreased total spending, since few people who have access to them use them to browse for lower-cost services [7,11].

In a recent paper, researchers addressed 1 possible reason for low engagement—lack of awareness. They implemented an extensive targeted online advertising campaign using Google Advertisements to increase awareness and assessed whether it increased New Hampshire’s PT website use. Their findings suggested that although lack of awareness is a possible reason for the low impact of PT tools in health care spending, structural factors might affect the use of health care information [12]. Individuals may not be able to exactly determine their out-of-pocket expenses from the information provided.

Surprisingly, there is little research on the awareness and usability of PT information after the current PT legislation went into effect. A recent study [13] highlighted the nonusability of existing machine-readable files for employers, policy makers, researchers, or consumers, and this paper adds to this literature by answering the question—is PT information as provided usable for patients or machines? Clearly, if it is of value to patients, it can be useful; the reason to take the perspective of machines was to examine whether this information as provided might also be useful for third-party programs that can extract information from the provided data (to subsequently help patients through other ways of presenting this information perhaps). We address this question through a combination of user experiments and data schema analysis. While there are recent papers that have also argued that PT data have deficiencies [13,14], ours is the first to combine user experiments with analysis of data schema from several hospitals in Florida to make a combined claim on value for patients and machines. We hope this can add to the discourse on PT and what needs to be done to extract value for patients and the health care system as a whole.

### Background

#### Impact of PT Tools

The impact of PT tools on consumers and health care facilities has been investigated in the literature. Some studies showed that consumers with access to PT tools are more likely to reduce forgone needed services over time. Moreover, consumers who use tools tend to find the lowest service prices [8,15-17]. A few studies investigated the impact of PT tools on the selection of health care facilities. They illustrated that some consumers tend to change health care facilities pursuing lower prices, while some others prefer to stay with expensive ones, although they are aware of some other facilities that offer lower prices [9,18]. Finally, some research studied the impact of PT tools on cost and showed that some consumers experienced no effect, while others experienced decreases in average consumer expenses [8,17,18]. However, the impact of PT tools on health care facilities is inconclusive, meaning different studies concluded different effects. Some stated that PT tools decrease the prices of imaging and laboratory services, while others said that although public charge disclosure lowers health care facility charges, the final prices remained unchanged [17,18].

#### Legislation Related Works

In a study, researchers considered 20 leading US hospitals to assess provided chargemasters to understand to what extent patients can obtain information from websites to determine the out-of-pocket costs [19]. Their findings showed that although all hospitals provided chargemasters on their websites, they rarely offered transparent information, making it hard for patients to determine out-of-pocket costs. Their analysis used advanced diagnostic imaging services to assess hospitals’ chargemasters since these are the most common services people look for. Mehrotra et al [7] also mentioned that the most common searches belonged to outpatient visits, magnetic resonance imaging (MRI), and emergency department visits. To this end, we used “MRI scan of the brain before and after contrast” as one of the shoppable services in our analysis. Another study examined imaging services in children’s hospitals (n=89), restricting the analysis to hospitals (n=35) that met PT requirements—published chargemaster rates, discounted cash prices, and payer-negotiated prices in a machine-readable file, and published costs for 300 common shoppable medical services in a consumer-friendly format. Their study revealed that, in addition to a broad range of imaging service charges, most hospitals lack the machine-readable file requirement [20].

Arvisais-Anhalt et al [21] identified 11 hospitals with available chargemasters in Dallas County to compare the prices of a wide range of available services. They observed significant variations for a laboratory test: partial thromboplastin time, a medication: 5 mg tablet of amlodipine, and a procedure: circumcision. Reddy et al [22] focus on New York State to assess the accessibility and usability of hospitals’ chargemasters from patients’ viewpoint. They found that 189 out of 202 hospitals had a locatable chargemaster on their home page. However, only 37 hospitals contain the Current Procedural Terminology (CPT) code, which makes those without the CPT code unusable due to the existence of many different descriptions for the same procedure; for example, an elective heart procedure had 34 entries. We add to this considerable literature by examining a subset of Florida hospitals.

In a competitive market, higher-quality goods and services require higher prices [23]. Based on this, Patel et al [24] examined the relationship between the Diagnosis-Related Group (DRG) chargemaster and quality measures. Although prior research found no convincing evidence that hospitals with greater costs also delivered better care [25], they discovered 2 important quality indicators that were linked to standard charges positively and substantially—mortality rate and readmission rates—which both are quality characteristics that are in line with economic theory. Moreover, Patel et al [24] studied the variety of one of the most commonly performed services (vaginal delivery) as a DRG code, which motivated us to select “Vaginal delivery without sterilization or D&C without CC/MCC” as another shoppable service in our analysis.

## Methods

### Ethical Considerations

All data used in this study, including the secondary data set obtained from hospitals’ websites and the data collected during the user experiment, underwent a thorough anonymization process. The study was conducted under protocols approved by the University of South Florida institutional review board (STUDY004145: “Effect of price transparency regulation (PTR) on the public decisions”) under HRP-502b(7) Social Behavioral Survey Consent. This approval encompassed the use of publicly available anonymized secondary data from hospitals’ websites, as well as a user experiment aimed at assessing awareness of the PT rule and the usability of hospitals’ files. No individual-specific data were collected during the experiment, which solely focused on capturing subjects’ awareness and opinions regarding the PT rule and associated files. At the onset of the experiment, participants were provided with a downloadable consent form and were allowed to withdraw their participation at any time. Survey participants were offered a US $2 reward, and their involvement was entirely anonymous.

### Data Collection

According to CMS, “Starting January 1, 2021, each hospital operating in the United States will be required to provide clear, accessible pricing information online about the items and services they provide in two ways: 1- As a comprehensive machine-readable file with all items and services. 2- In a display of shoppable services in a consumer-friendly format.” As stated, files available on hospitals’ websites should be consumer-friendly, so the question of whether these files are for users arises. On the other hand, as stated, files should be machine-readable, so again the question of whether these files are for machines arises. Below we try to answer both questions in detail, respectively.

### Value for Users: User Experiments

#### Overview

When a public announcement is disseminated, its efficacy relies on ensuring widespread awareness and facilitating practical use during times of necessity. Previous research on PT announcements has highlighted the challenges faced by patients in accurately estimating out-of-pocket expenses. However, a fundamental inquiry arises—are individuals adequately informed about the availability of tools that enable them to estimate their out-of-pocket costs for desired services? To address this, we conducted a survey to assess public awareness of PT legislation. The survey encompassed a range of yes or no and multiple-choice questions aimed at gauging participants’ familiarity with the PT rule in health care and their entitlement to obtain cost information prior to receiving a service. Additionally, we inquired about participants’ knowledge of resources for accessing pricing information and whether they were aware of the PT rule. Furthermore, we incorporated follow-up questions to ensure that the survey responses were not provided arbitrarily, thereby securing reliable and meaningful outcomes.

Moreover, considering the previously established evidence of subpar usability associated with the currently available files, we propose streamlining the existing files and developing a user-friendly and comprehensive document for conducting an A/B test. This test aims to evaluate which file better facilitates participants in accurately estimating their out-of-pocket costs. In collaboration with Florida Blue experts during biweekly meetings throughout the entire process outlined in this paper, the authors determined the optimal design for the summary table. This design, which presents prices in a more user-friendly format, enhancing overall participant comprehension, was used during the A/B testing. Participants were randomly assigned to either access the hospitals’ files or a meticulously constructed summary table, manually created in Excel, prominently displaying cost information (Please note that all files, including the hospitals’ files and our Excel file, are made available in the same format [Excel] on a cloud-based platform to eliminate any disparities in accessing the files. This ensures equitable ease of finding, downloading, and opening files, as accessing the hospitals’ files typically requires significant effort.). The experiment entailed presenting 3 distinct health-related scenarios and instructing participants to locate the price for the requested service. Subsequently, participants were asked to provide the hospital name, service price, insurer name, and insurance plan. Additionally, we sought feedback on the perceived difficulty of finding the requested service and their priority for selecting hospitals [26], followed by Likert scale questions to assess participants’ evaluation of the provided file’s efficacy in facilitating price retrieval.

The experiments were conducted to investigate the following questions: (1) Are the individuals aware of the PT legislation? and (2) Is the information provided usable for patients? To evaluate the usability of files found on websites, we selected 2 prevalent services based on existing literature and 2 other services recommended as high-demand ones by Florida Blue experts, Table 1. Furthermore, meticulous efforts were made to ensure that both the control and treatment groups encountered identical circumstances, thus allowing for a systematic examination of the disparities solely attributable to variations in data representation.

**Table 1:** Detail information on selected services for the experiment.

Service code	Code type	Service full name	References
807	DRG^a^	Vaginal delivery without sterilization or D&C^b^ without CC/MCC^c^	[24]
29827	CPT^d^	Arthroscopic cuff repair (shoulder arthroscopy)	Suggested by Florida Blue experts as high-demand services.
45380	CPT	Colonoscopy, flexible, with biopsy, single, or multiple	Suggested by Florida Blue experts as high-demand services.
70553	CPT	MRI^e^ scan of brain before and after contrast	[7,20]

^a^DRG: Diagnosis-Related Group.

^b^D&C: dilation and curettage.

^c^CC/MCC: complication or comorbidity/major complication or comorbidity.

^d^CPT: Current Procedural Terminology.

^e^MRI: magnetic resonance imaging.

#### Participants

A total of 67 adults (30 female individuals; mean 41.43, SD 12.39 years) were recruited on the Amazon Mechanical Turk platform, with no specific selection criteria other than being located in the United States.

#### Data

We focused on 75 main hospitals (ie, the main hospital refers to distinguish a hospital from smaller clinics or specialized medical centers within the same health system) in the state of Florida. When we searched their websites for PT files (machine-readable files), only 89% (67/75) of hospitals included machine-readable files. According to the PT legislation, these files were supposed to contain information about 300 shoppable services. However, only 58% (39/67) of hospitals included information such as insurer prices in their files. Therefore, for the rest of the analysis, we only included the 39 hospitals that have the required information in their machine-readable files on their websites. We created an Excel file that included those 39 hospitals along with the 4 services—CPT 45380, 29827 and 70553 and DRG 807—mentioned in the literature (Table 1) for 4 popular (suggested by Florida Blue experts) commercial carriers (Health Maintenance Organization [HMO] or Preferred Provider Organization [PPO] plans)—Aetna, Florida Blue, Cigna, and UnitedHealthcare.

#### Procedure

Participants were recruited for the pilot and randomly assigned by the Qualtrics XM platform to answer multiple-choice questions and fill in blanks based on the given scenarios. First, participants responded to questions regarding the awareness of PT and then were divided into 2 groups randomly to answer questions regarding the usability of hospital-provided PT information. One group was assigned hospitals’ website links (control group), while the other group was given an Excel file with the same information provided in files on hospitals’ websites, but in a manner that was designed to allow easier comparison of prices across hospitals (Multimedia Appendix 1). Participants were given 3 scenarios that asked them to find a procedure’s price based on their hospital and insurer selection to compare hospital-provided information with Excel. We provide some examples of hospitals’ files and our Excel file in Multimedia Appendix 1 and the survey experiment questions in Multimedia Appendix 2*.*

### Value for Machines: Schema Integration—Machine-Readable Files Representation

Through meticulous investigation of machine-readable files from 39 hospitals, we discovered that these files may vary in formats such as CSV or JSON, posing a challenge for machines to effectively manage the data within these files. Another significant obstacle arises from the lack of uniformity in data representation across these files, rendering them unsuitable for machine use without a cohesive system capable of processing them collectively. Our analysis revealed that hospitals within a single health system exhibit consistent data representation, although service prices may differ (we include both the same and different chargemaster prices in our study), while substantial disparities in data representation exist between hospitals affiliated with different health systems.

Moving forward, we will use the terms “data representation” and “schema” interchangeably, with “schema” denoting its database management context. In this context, a schema serves as a blueprint outlining the structure, organization, and relationships of data within a database system. It encompasses key details such as tables, fields, data types, and constraints that define the stored data. To systematically illustrate schema differences among hospitals associated with different health systems, we adopted the methodology outlined in reference [27] for schema integration, which offers a valid approach for comparing distinct data representations. The concept of schema integration encompasses four common categories: (1) identical: hospitals within the same health system adhere to this concept as their representations are identical; (2) equivalent: while hospitals in health system “A” may present different representations from those in health system “B,” they possess interchangeable columns; (3) compatible: in cases where hospitals across different health systems are neither identical nor equivalent, the modeling constructs, designer perception, and integrity constraints do not contradict one another; and (4) incompatible: in situations where hospitals within different health systems demonstrate contradictory representations, distinct columns exist for each health system due to specification incoherence.

Our analysis focused on health systems in Florida that encompassed a minimum of 4 main hospitals, using the most up-to-date data available on their respective websites. Within this scope, we identified 8 health systems with at least 4 main hospitals, of which 88% (7/8) of health systems had published machine-readable files on their websites. Consequently, our analysis included 65% (36/55) of hospitals that possessed machine-readable files available on their websites. To facilitate further investigation by interested researchers, we have made the analyzed data accessible on a cloud-based platform. During our analysis, we meticulously extracted the schema of each health system by closely scrutinizing the hospitals associated with each health system, capturing key details such as tables, fields, and data types. Subsequently, we compiled a comprehensive master field name table trying to have the same data type and field names that make it easier for machines to retrieve information. We elaborate on the master field names table in greater detail within the results section.

## Results

### Value for Users

#### Question 1 (PT Awareness)

Based on the responses, it is evident that participants need to be made aware of the PT legislation. Among the participants, 64% (49/76) reported that they had not heard about the legislation. However, they believe it is important to know the service price before receiving it—response charts are provided in Multimedia Appendix 3.

#### Question 2 (Human Usability of PT Information)

Based on the responses to scenarios, the average number of correct responses is not equal between the 2 groups, that is, the treatment group (mean 1.23, SD 1.30) found more correct answers than the control group (mean 2.76, SD 0.58; *t*_65_=6.46; *P*<.049; *d*=1.52). The *t* tests (2-tailed) for the other questions in the experiment are in Multimedia Appendix 4.

These suggest that current files on hospitals’ websites are not consumer-friendly, and participants find it challenging to estimate out-of-pocket costs for a desired service. For this reason, in addition to making the files easier to use, this information should also include thorough documentation that explains what each column represents, up to what amount an insurer covers for a specific service, or the stated price covers up to how many days of a particular service, that is, “contracting method.” For example, based on consulting with one of the senior network analysts of Florida Blue, some prices for a service like DRG 807 are presented as per diem costs, and based on the current information on these files, it cannot be recognizable without having comprehensive documentation for them.

### Value for Machines

After carefully reviewing all machine-readable file schemas, we create a master field name table, including the available field names in machine-readable files (Table 2). According to Table 2, the first column represents master field names that we came up with, and the following columns each represent hospitals within a health system. The “✓” mark shows that hospitals within a health system have identical field names as we consider as master field names and the “written” cells show equivalent field names, meaning that hospitals within that health system use different field names—we write what they use in their representation—while the content is equivalent to what we select as the master field name. The “❋” mark means that although hospitals within health system #2 provide insurer names and plans in their field names, some codes make those columns unusable for machines to recognize them the same as master field names. We also include the type of field names for all representations in parentheses.

**Table 2:** Master field names that can be used for all hospitals within all health systems.

Master field names^a^	Hospitals within health system #1	Hospitals within health system #2	Hospitals within health system #3	Hospitals within health system #4	Hospitals within health system #5	Hospitals within health system #6	Hospitals within health system #7
Code | (type)	✓^b^ | (str^c^)	✓ | (int^d^)	✓ | (int)	Final CPT^e^ | (str)	✓ | (str)	HCPCS^f^/CPT Code| (str)	CPT code/HCPCS | (str)
Description | (type)	✓ | (str)	✓ | (str)	✓ | (str)	Tech name | (str)	Code description | (str)	✓ | (str)	Procedure description | (str)
Code type | (type)	✓ | (str)	✓ | (str)	—^g^	—	✓ | (str)	—	—
Apr^h^ DRG^i^ | (type)	—	—	—	✓ | (int)	—	—	✓ | (int)
Ms^j^ DRG | (type)	—	—	—	✓ | (int)	—	—	✓ | (int)
Ms DRG Title | (type)	—	—	—	✓ | (str)	—	—	—
CDM^k^ service description | (type)	—	—	—	—	—	—	✓ | (str)
Patient class | (type)	Type | (str)	—	Type | (str)	—	✓ | (str)	—	—
Revenue description | (type)	—	UB^l^ revenue description | (str)	—	Final rev | (str)	—	—	—
Revenue code | (type)	Rev code | (int)	UB revenue code | (int)	—	Final rev | (int)	—	—	✓ | (int)
Package or line level | (type)	—	—	✓ | (str)	—	—	—	—
Procedure ID | (type)	—	—	—	Srvc Prvd Srvc ID | (int)	—	✓ | (int)	Procedure code/charge code | (int)
Payer | (type)	—	—	—	—	✓ | (str)	—	—
Payer specific negotiated charge | (type)	—	—	—	—	✓ | (float^m^)	—	—
Gross charge | (type)	✓ | (float)	✓ | (float)	✓ | (float)	✓ | (float)	✓ | (float)	✓ | (float)	Default charge | (int)
Discounted cash price | (type)	✓ | (float)	Cash charge | (float)	Derived contracted rate | (float)	✓ | (float)	✓ | (float)	✓ | (float)	Cash charge | (float)
Min negotiated rate | (type)	Min | (float)	✓ | (float)	Deidentified min contracted rate | (float)	✓ | (float)	Deidentified min contracted charge | (float)	—	Minimum | (int)
Max negotiated rate | (type)	Max | (float)	✓ | (float)	Deidentified max contracted rate | (float)	✓ | (float)	Deidentified max contracted charge | (float)	—	Maximum | (int)
Self pay | (type)	—	—	✓ | (float)	✓ | (float)	—	—	—
Insurer name | (type)	✓ | (str)	❋^n^ | (str)	✓ | (str)	✓ | (str)	—	—	✓ | (str)
Insurer plan | (type)	✓ | (str)	❋ | (str)	✓ | (str)	✓ | (str)	—	—	✓ | (str)
Price | (type)	✓ | (float)	✓ | (float)	✓ | (float)	✓ | (float)	—	—	✓ | (float)

^a^As noted previously, since we focus on the health system level instead of the hospital level, our schema does not have hospital-level information; however, it would be beneficial to add hospital information to the table.

^b^✓: it means the given master field name in that row appears on the given health system file in that column.

^c^str: shows “string” as the data type.

^d^int: shows “integer” as the data type.

^e^CPT: Current Procedural Terminology.

^f^HCPCS: Health care Common Procedure Coding System.

^g^Not applicable.

^h^Apr: all patients refined.

^i^DRG: Diagnosis-Related Group.

^j^Ms: Medicare severity.

^k^CDM: charge description master.

^l^UB: uniform billing.

^m^float: it shows “float” as the data type.

^n^❋: it means that although hospitals within health system #2 provide insurer names and plans in their field names, some codes make those columns unusable for machines to recognize them the same as master field names.

**Figure 1:**
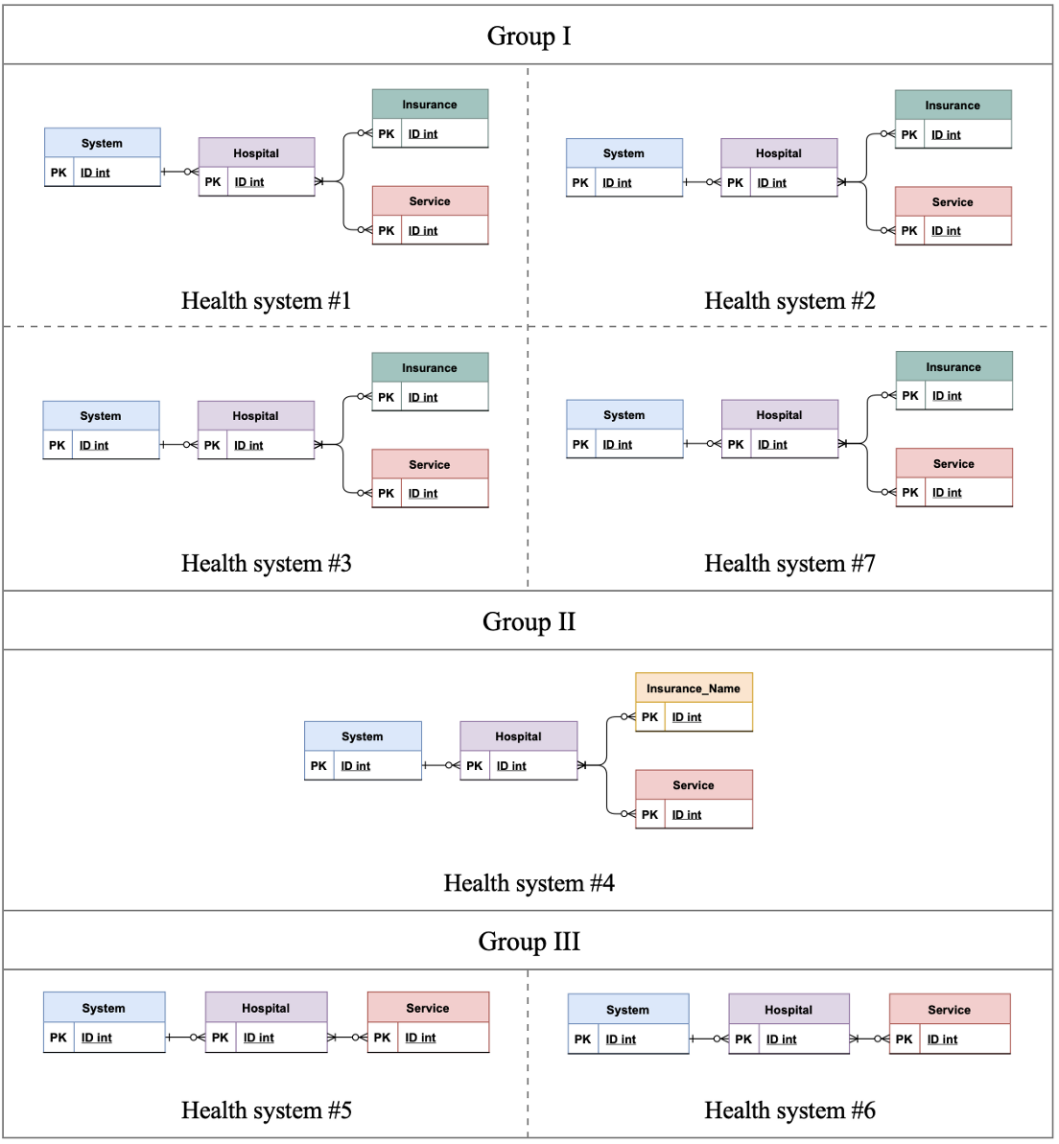
Grouping hospitals’ ERDs within each health system. ERD: entity-relationship diagram; PK: primary key.

We did reverse engineering and drew entity-relationship diagrams (ERDs) for each hospital based on their data representation. However, as hospitals within the same health system have the same ERDs, we only include 1 ERD for each health system (Figure 1). According to Figure 1, although hospitals have tried to follow an intuitive structure, we can still separate them into three groups: (1) group I: all hospitals within this group have several columns for different insurers. As shown in the ERDs, we decided to have a separate entity, called “Insurance” for this group; (2) group II: all hospitals within this group have many sheets, and each sheet belongs to a specific insurer with a specific plan. As shown in the ERDs, we decided to create an “Insurance_Name” entity for this group’s ERD to show the difference in data representation; and (3) group III: all hospitals within this system have a “payer” column which includes the names of insurers without their plans. As shown in the ERDs, we decided to put this column as an attribute in the “Service” entity, and do not have an “Insurance” entity for this group’s ERD.

In conclusion, although most hospitals have adopted group I logic for data representation, for full similarity, a standard representation with the same intuitive field names (like what we suggest as the master field name; Table 2) should be proposed so that it can cover all systems’ data representations and be used as machine-readable file, for at least machine benefits. Mainly, standardization in the format and semantics of the provided data can help substantially in making the data more machine friendly.

## Discussion

### Comparison With New CMS Guidelines

Recently, CMS has published guidelines regarding the PT legislation [28]. The most recent CMS guideline is a step forward in ensuring standardization but is still only recommended and is not mandatory. These guidelines exhibit overlaps with our fields in Table 2, with slight differences attributed to granularities. Our observation reveals that hospitals within the same health system adopt a uniform schema. Therefore, our suggested schema operates on the granularity of health systems rather than individual hospitals.

The recent CMS guidelines allocate 24% (6/25) of field names specifically to hospital information, encompassing details such as “Hospital Name,” “Hospital File Date,” “Version,” “Hospital Location,” “Hospital Financial Aid Policy,” and “Hospital Licensure Information.” These details, absent in current hospital files, are crucial for informed decision-making. As noted previously, since we focus on the health system level instead of the hospital level, our schema does not have hospital-level information; however, it would be beneficial to add hospital information to the tables.

Our analysis reveals that the 11 field names in Table 2 align with the field names in the new CMS guidelines, demonstrating a substantial overlap of 58% (11/19). The corresponding CMS field names (compatible with our schema) include “Item or Service Description (Description or CDM Service Description),” “Code (Code),” “Code Type (Code Type),” “Setting (Patient Class),” “Gross Charge (Gross Charge),” “Discounted Cash Price (Discounted Cash Price),” “Payer Name (Insurer Name),” “Plan Name (Insurer Plan),” “Payer Specific Negotiated Charge: Dollar Amount (Price),” “De-identified Minimum Negotiated Charge (Min Negotiated Rate),” and “De-identified Maximum Negotiated Charge (Max Negotiated Rate).” Additionally, both our schema and the new CMS guidelines propose data types for each field name.

In our schema, which represents current hospitals’ files, there are 5 field names absent in the new CMS guidelines “Revenue Description,” “Revenue Code,” “Package/Line Level,” “Procedure ID,” and “Self Pay.” Conversely, the new CMS guidelines introduce 8 additional field names “Billing Class,” “Drug Unit of Measurement,” “Drug Type of Measurement,” “Modifiers,” “Payer Specific Negotiated Charge: Percentage,” “Contracting Method,” “Additional Generic Notes,” and “Additional Payer-Specific Notes.” We regard these new field names as providing further detailed information and enhancing consumer decision-making. If hospitals within a health system adopt consistent formats and can map their formats to the new CMS guidelines clearly in a mapping document they also provide, this can be more useful than the current optional guideline that is suggested.

In summary, since our analysis is based on the current data schema that hospitals have in place, we believe the schema we put out is easier to implement with minimal change to what the hospitals are currently doing. However, given the recent CMS guidelines, we recommend adding 8 additional fields as well as hospital-specific information.

### Implications

The PT legislation aims to enable informed decision-making, reduce out-of-pocket expenses, and decrease overall health care expenditures. This study investigates the usage of current files by individuals and machines. Our results, unfortunately, suggest that PT data—as currently reported—appear to be neither useful for patients nor machines, raising important questions as to what these appear to be achieving today. Moreover, the findings indicate that even individuals with basic computer knowledge struggle with the usability of these files, highlighting the need for significant revisions to make them consumer-friendly and accessible to individuals of all technical proficiency levels. Additionally, inconsistencies in data representation between hospitals affiliated with different health systems pose challenges for machines, necessitating schema design improvements and the implementation of a standardized data representation. By addressing these concerns, PT legislation can achieve consistency and enhance machine readability, thus improving its effectiveness in promoting informed decision-making and reducing health care costs.

Although the official announcement of PT legislation is recent, prior studies [15-17] have attempted to evaluate the usability of PT, while subsequent studies [19-22] have examined the effectiveness of PT tools following the announcement. However, despite the introduction of PT rules, it appears that the usability of these files has not undergone significant improvements, indicating the necessity for proactive measures from responsible executives to ensure the effectiveness of this legislation. Our analysis of this matter emphasizes 2 primary factors—a lack of awareness among stakeholders and the challenges associated with using files due to inconsistencies in their format and representation.

As of April 2023, the CMS has issued over 730 warning notices and 269 requests for Corrective Action Plans. A total of 4 hospitals have faced Civil Monetary Penalties for noncompliance, and these penalties are publicly disclosed on the CMS website. The remaining hospitals subjected to comprehensive compliance reviews have either rectified their deficiencies or are actively engaged in doing so. While we acknowledge these efforts to comply with PT rules, our research revealed a notable disparity in data representation among hospitals affiliated with different health systems. Consequently, we focused on schema design and proposed the implementation of a master field name that encompasses a comprehensive data representation derived from an analysis of 36 hospitals. Standardizing the data representation across all health systems’ machine-readable files will effectively address concerns about consistency. Therefore, significant modifications are required for the PT legislation to enhance machine readability and provide clearer guidance on the design and structure of the files’ schema. If the hospital-provided information is consistent and of high quality, PT tools provided by health insurers may be able to estimate an individual’s total expenses more accurately.

### Limitations

Our objective was to have an equal number in both groups. However, in the case of the group tasked with obtaining information from the hospitals’ websites, most did not finish the task and dropped out without completing it. This occurred because the task of retrieving the cost from the hospitals’ websites in its current form is complex, as indicated by feedback from some participants. Only 19% (13/67) completed the task in that group (control group). Although this is a limitation of the study, it also highlights the complexity of obtaining cost information from hospitals’ websites in the current form. In the treatment group, 81% (54 out of 67) of participants completed the task of retrieving the data, and the completion percentage was much higher.

### Conclusions

Due to the poor usability and inconsistency of the formats, we, unfortunately, did not find evidence that the PT rule as implemented currently is useful to consumers, researchers, or policy makers (despite the legislation’s goals that files are “consumer-friendly” and “machine-readable”). As 1 solution, we suggest a master field name for the data representation of machine-readable files to make them consistent, at least for the machines. Building tools that enable customers to estimate out-of-pocket costs is facilitated by having consistent machine-readable files across all health systems, which can be considered as future work for researchers and companies to help the PT rule reach its main goal, which is providing useful information for patients and reducing health care expenditures. In addition, another worthwhile approach to reducing some of the exorbitant health care costs in the United States would be to integrate clinical decision support tools into the providers’ workflow, triggered by orders for medications, diagnostic testing, and other billable services. In this regard, Bouayad et al [29] conducted experiments with physicians to demonstrate that PT, when included as part of the system they interact with, such as clinical decision support integrated into electronic health record systems, can significantly aid in cost reduction. This is a promising direction for practice but needs to be implemented carefully to avoid unanticipated consequences, such as scenarios where cost is incorrectly viewed as a proxy for quality, or where the use of this information introduces new biases for physicians and patients.

## Appendix

Multimedia Appendix 1 Example of Excel format of hospitals’ files and our created Excel file.

Multimedia Appendix 2 Survey questions and experiment scenarios.

Multimedia Appendix 3 Participants’ responses chart regarding price transparency awareness.

Multimedia Appendix 4 The *t* test analysis regarding human usability of price transparency information based on participants’ responses.

## Abbreviations

CMS: Centers for Medicare & Medicaid Services
CPT: Current Procedural Terminology
DRG: Diagnosis-Related Group
ERD: entity-relationship diagram
HMO: Health Maintenance Organization
MRI: magnetic resonance imaging
PPO: Preferred Provider Organization
PT: price transparency
PTR: price transparency regulation
