# Supplementary material for: Usability of Health Care Price Transparency Data in the United States: Mixed Methods Study
Source: J Med Internet Res. 2024 Mar 29;26:e50629. doi: 10.2196/50629 (PMC11015359; doi:10.2196/50629)
Supplement: Multimedia Appendix 1 [file jmir_v26i1e50629_app1.pdf]

Multimedia Appendix 1: Example of Excel format of hospitals’ files and our created Excel file

Example of Excel format of hospitals’ files:

	Code	Description	Type	Package/Line_Level	Gross Charge	De-identified min contracted rate	De-identified max contracted rate	Derived contracted rate	Self-Pay	Aetna-Commercial HMO/POS_Avg	Aetna-Commercial PPO/Open Access_Avg	Aetna-Medicare Advantage HMO_Avg
2	120	Anesthesia f	Outpatient	Line	4585	2874.15	3672	3158.38	2751	0	0	0
3	160	Anesthesia f	Outpatient	Line	2929	2929	4131	3530	1757.4	0	0	0
4	320	Anesthesia f	Outpatient	Line	1687	11.5	162345	1561.82	1012.2	1589.65	0	0
5	520	Anesthesia f	Outpatient	Line	1687	918	2101	1585.18	1012.2	0	0	0
6	530	Anesthesia f	Outpatient	Line	1273	639.05	3757	2198.03	763.8	639.05	0	0
7	532	Anesthesia f	Outpatient	Line	2515	1836	2101	1968.5	1509	0	0	0
8	630	Anesthesia f	Outpatient	Line	3550	3343	3343	3343	2130	0	0	0
9	670	Anesthesia f	Outpatient	Line	5413	1818.14	5967	3894.03	3247.8	0	0	0
10	731	Anesthesia f	Outpatient	Line	1080.87	125.41	2295	1193.82	648.522	1741.73	0	125.41
11	732	Anesthesia f	Outpatient	Line	1687	2101	2754	2456.67	1012.2	0	0	0
12	790	Anesthesia f	Outpatient	Line	1819.83	2101	2929	2515	1091.898	2101	0	0
13	811	Anesthesia f	Outpatient	Line	1355.8	506.1	1687	1199.53	813.48	0	0	0
14	840	Anesthesia f	Outpatient	Line	2751.57	912.38	6426	2958.78	1650.942	0	0	0
15	902	Anesthesia f	Outpatient	Line	1273	543.05	543.05	543.05	763.8	0	0	0
16	910	Anesthesia f	Outpatient	Line	1273	1273	1687	1514.67	763.8	0	0	0
17	918	Anesthesia f	Outpatient	Line	1687	1687	2754	2156.43	1012.2	0	0	0
18	940	Anesthesia f	Outpatient	Line	2308	17.31	4908	264.69	1384.8	425.12	939	0
19	952	Anesthesia f	Outpatient	Line	1687	446.61	2101	1728.56	1012.2	0	0	446.61
20	1250	Anesthesia f	Outpatient	Line	3136	306.75	4171	2850.69	1881.6	0	0	306.75
21	1400	Anesthesia f	Outpatient	Line	1687	1273	3757	2367.75	1012.2	0	0	0
22	1402	Anesthesia f	Outpatient	Line	3964	2929	3757	3481	2378.4	0	0	0
23	1480	Anesthesia f	Outpatient	Line	2515	1687	3213	2195.67	1509	0	0	0
24	1844	Anesthesia f	Outpatient	Line	6655	1594.19	5967	3772.73	3993	0	0	0
25	1922	Anesthesia f	Outpatient	Line	2101	15.69	4171	1741.83	1260.6	0	0	0
26	1965	Anesthesia f	Outpatient	Line	1635.25	693.05	2101	1398.42	981.15	0	0	0
27	1967	Anesthesia f	Outpatient	Line	818	438.8	438.8	438.8	490.8	438.8	0	0
28	10005	Fine needle	Outpatient	Line	1216.7	177.24	2766	997.44	730.02	1203.29	579.46	579.46
29	10006	Fine needle	Outpatient	Line	932.96	120.45	1638.58	713.63	559.776	1638.58	0	120.45
30	10035	Placement o	Outpatient	Line	1772	608.43	994.09	742.68	1063.2	0	0	0
31	10060	Drainage of	Outpatient	Line	861	83.64	861	372.46	516.6	726.68	167.28	0
32	10061	Drainage of	Outpatient	Line	1666.04	69.33	9785	839.67	999.624	1281.74	1345.9	322.21
33	10080	Drainage of	Outpatient	Line	2518	110.27	2062.24	914.5	1510.8	2062.24	0	0
34	10081	Drainage of	Outpatient	Line	4180	579.46	4180	2429.49	2508	3176.8	0	0
35	10120	Removal of f	Outpatient	Line	1363	1363	5711.56	2812.52	817.8	5711.56	0	0
36	10160	Aspiration of	Outpatient	Line	962	110.27	1936	490.06	577.2	0	0	322.21
37	11010	Removal of f	Outpatient	Line	4192	750.06	1613.92	1181.99	2515.2	750.06	0	0
38	11012	Removal of f	Outpatient	Line	2675.34	629.09	629.09	629.09	1605.204	0	0	0
39	11042	Removal of s	Outpatient	Line	1205.21	55.09	10796	804.73	723.126	1124.49	972.42	326.46
40	11043	Removal of s	Outpatient	Line	3051.17	253.54	5725.33	1892.01	1830.702	2288.11	488.35	490.43
41	11044	Removal of s	Outpatient	Line	4457	1376.39	6452.15	3736.47	2674.2	0	0	0
42	11045	Removal of s	Outpatient	Line	520.76	90.72	9072	498.03	312.456	1008	0	0
43	11046	Removal of s	Outpatient	Line	1044	325.2	9396	984.47	626.4	0	0	0

	Code	Amerigroup-Medicare Advantage HMO_Avg	Blue Cross-Commercial PPO/Open Access_Avg	Avmed-Commercial HMO/POS_Avg	BayCare Select-Commercial HMO/POS_Avg	Cigna-Commercial PPO/Open Access_Avg	Careplus-Medicare Advantage HMO_Avg	Cigna-Commercial HMO/POS_Avg	Devoted Health-Medicare Advantage HMO_Avg	Blue Cross-Commercial other_Avg	United Healthcare-Commercial PPO/Open Access_Avg	Blue Cross-Commercial HMO/POS_Avg	Humana-Commercial HMO/POS_Avg	Humana-Medicare Advantage HMO_Avg	Humana-Commercial PPO/Open Access_Avg
2	120	0	0	0	0	0	0	0	0	0	0	0	0	0	0
3	160	0	0	0	0	0	0	0	0	0	0	0	0	0	0
4	320	0	0	1450.07	0	0	264.81	4046.18	0	2791.39	0	1147.2	0	144.16	0
5	520	0	0	0	0	0	0	0	0	0	0	979	0	0	0
6	530	0	0	0	0	0	0	0	0	0	0	0	0	0	0
7	532	0	0	0	0	0	0	0	0	0	0	0	0	0	0
8	630	0	0	0	0	0	0	0	0	0	0	0	0	0	0
9	670	0	0	0	0	0	0	0	0	2929	0	1818.14	0	0	0
10	731	0	0	0	0	0	0	0	0	0	0	0	0	0	0
11	732	0	0	0	0	0	0	0	0	0	0	0	0	0	0
12	790	0	0	0	0	0	0	0	0	0	0	0	0	0	0
13	811	0	0	0	0	0	0	0	506.1	0	0	0	0	0	0
14	840	0	0	0	0	0	0	3040.35	1002.9	0	0	1514.2	0	0	0
15	902	0	0	0	0	0	0	0	0	0	0	543.05	0	0	0
16	910	0	0	0	0	0	0	0	0	0	0	0	0	0	0
17	918	0	0	0	0	0	0	0	0	0	0	0	0	0	0
18	940	0	0	0	0	0	80.34	254.15	0	548.11	0	660.68	0	0	0
19	952	0	0	0	0	0	0	0	0	0	0	0	0	0	0
20	1250	0	0	0	0	0	0	0	0	0	0	0	0	0	0
21	1400	0	0	0	0	0	0	0	0	0	0	0	0	0	0
22	1402	0	0	0	0	0	0	0	0	3757	0	0	0	0	0
23	1480	0	0	0	0	0	0	0	0	3213	0	0	0	0	0
24	1844	0	0	0	0	0	0	0	0	0	0	1594.19	0	0	0
25	1922	0	0	0	0	0	0	0	0	0	0	0	0	0	0
26	1965	0	0	0	0	0	0	0	0	0	0	729.8	0	0	0
27	1967	0	0	0	0	0	0	0	0	0	0	0	0	0	0
28	10005	0	0	635.04	580.84	0	0	467.39	364.2	559.38	0	528.88	2397	0	0
29	10006	0	0	0	0	0	0	0	247.5	674.8	0	426.51	0	0	0
30	10035	0	0	0	0	0	0	994.09	0	0	0	0	0	0	0
31	10060	0	0	0	167.28	0	0	483.04	0	325.84	0	315.24	0	236.87	0
32	10061	0	0	0	0	0	0	877.96	0	839.91	0	678.1	0	456.25	0
33	10080	0	0	0	0	0	0	0	0	1319.68	0	0	0	0	0
34	10081	0	0	0	0	0	0	2232.12	0	0	0	0	0	0	0
35	10120	0	0	0	0	0	0	0	0	0	0	0	0	0	0
36	10160	0	0	0	161.11	0	0	370.37	0	405.39	0	0	0	0	0
37	11010	0	0	0	0	0	0	1613.92	0	0	0	0	0	0	0
38	11012	0	0	0	0	0	0	0	0	0	0	0	0	0	0
39	11042	0	0	613.7	322.55	0	312.16	557.92	0	495.5	338.32	522.23	1490.88	457.2	1887.67
40	11043	0	0	0	488.77	0	774.54	1188.22	893.7	1577.53	0	1098.88	0	0	0
41	11044	0	0	0	0	0	0	0	0	4612.68	0	1388.36	0	0	0
42	11045	0	0	0	0	0	134.04	194.04	0	540.97	0	214.68	0	0	676
43	11046	0	0	0	0	0	0	401.94	0	789.97	0	504.9	0	0	0

MortonPlantHospital

1	CDM SECTION									
2	ISSUER_ID_FACILITY	SRVC_PRVD_SRVC_ID	TECH_NAME	Final Rev	Final CPT	GROSS CHARGE	NEGOTIATED MINIMUM	NEGOTIATED MAXIMUM	DISCOUNTED CASH PRICE	FLORIDA BLUE, HOI-COMMERCIAL HMO
63613		37402377	Brain & stem MRI w wo con	611	70553	$ 6,030.00	$ 1,193.94	$ 5,427.00	$ 3,919.50	$ 3,618.00
63614		37402385	Brain & stem MRI w wo con ltd	611	70553	$ 6,030.00	$ 1,193.94	$ 5,427.00	$ 3,919.50	$ 3,618.00
63623		44003234	Brain & stem MRI w wo con	611	70553	$ 6,030.00	$ 1,193.94	$ 5,427.00	$ 3,919.50	$ 3,618.00
63630		91565788	Brain & stem MRI w wo con	611	70553	$ 6,030.00	$ 1,193.94	$ 5,427.00	$ 3,919.50	$ 3,618.00
63631		91565796	Brain & stem MRI w wo con ltd	611	70553	$ 6,030.00	$ 1,193.94	$ 5,427.00	$ 3,919.50	$ 3,618.00
69664										
69665										
69666	HCPCS Section	HCPCS Code	Charge Description			GROSS CHARGE	NEGOTIATED MINIMUM	NEGOTIATED MAXIMUM	DISCOUNTED CASH PRICE	FLORIDA BLUE, HOI-COMMERCIAL HMO
69667		10005	Fna bx w/us gdn 1st les			N/A	N/A	N/A	N/A	N/A
69668		10007	Fna bx w/fluor gdn 1st les			N/A	N/A	N/A	N/A	N/A
69669		10009	Fna bx w/ct gdn 1st les			N/A	N/A	N/A	N/A	N/A
69670		10011	Fna bx w/mr gdn 1st les			N/A	N/A	N/A	N/A	N/A
69671		10021	Fna bx w/o img gdn 1st les			N/A	N/A	N/A	N/A	N/A
69672		10030	Guide cathet fluid drainage			N/A	N/A	N/A	N/A	N/A
69673		10035	Perq dev soft tiss 1st imag			N/A	N/A	N/A	N/A	N/A
69674		10040	Acne surgery			N/A	N/A	N/A	N/A	N/A
69675		10060	Drainage of skin abscess			N/A	N/A	N/A	N/A	N/A
69676		10061	Drainage of skin abscess			N/A	N/A	N/A	N/A	N/A
69677		10080	Drainage of pilonidal cyst			N/A	N/A	N/A	N/A	N/A
69678		10081	Drainage of pilonidal cyst			N/A	N/A	N/A	N/A	N/A
69679		10120	Remove foreign body			N/A	N/A	N/A	N/A	N/A
69680		10121	Remove foreign body			N/A	N/A	N/A	N/A	N/A
69681		10140	Drainage of hematoma/fluid			N/A	N/A	N/A	N/A	N/A
69682		10160	Puncture drainage of lesion			N/A	N/A	N/A	N/A	N/A
69683		10180	Complex drainage wound			N/A	N/A	N/A	N/A	N/A
69684		11000	Debride infected skin			N/A	N/A	N/A	N/A	N/A
69685		11010	Debride skin at fx site			N/A	N/A	N/A	N/A	N/A
69686		11011	Debride skin musc at fx site			N/A	N/A	N/A	N/A	N/A
69687		11012	Deb skin bone at fx site			N/A	N/A	N/A	N/A	N/A
69688		11042	Deb subq tissue 20 sq cm/<			N/A	N/A	N/A	N/A	N/A
69689		11043	Deb musc/fascia 20 sq cm/<			N/A	N/A	N/A	N/A	N/A
69690		11044	Deb bone 20 sq cm/<			N/A	N/A	N/A	N/A	N/A
69691		11055	Trim skin lesion			N/A	N/A	N/A	N/A	N/A
69692		11056	Trim skin lesions 2 to 4			N/A	N/A	N/A	N/A	N/A
69693		11057	Trim skin lesions over 4			N/A	N/A	N/A	N/A	N/A
69694		11102	Tangntl bx skin single les			N/A	N/A	N/A	N/A	N/A

OP AVMED COMMERCIAL POS | OP AVMED ENTRUST EXCHANGE | OP FL BLUE HOI COMMERCIAL HMO | OP FL BLUE MYBLUE EXCHANGE | OP FL BLUE HOI SIMPLY BLU

1	file_create_date	run_id	name	tax_id	code	code type	code description	payer	patient_class	gross charge	de-identified minimum negotiated charge	payer-specific negotiated charge	de-identified maximum negotiated charge	discounted cash price	charge_code
2	2/18/22	d5077cc8-85		75-2922687	J7120	cpt	ringers lacta	aetna	I	711.41	58.7	N/A	298.06	533.56	
3	2/18/22	d5077cc8-85		75-2922687	S0028	cpt	injection, far	aetna	O	1149.61	99.77	268.8	456.34	862.21	
4	2/18/22	d5077cc8-85		75-2922687	P9021	cpt	red blood cel	aetna	O	467	104.37	104.37	133.52	350.25	
5	2/18/22	d5077cc8-85		75-2922687	Q9968	cpt	visualization	aetna	O	20.2	1.05	N/A	6.97	15.15	
6	2/18/22	d5077cc8-85		75-2922687	P9047	cpt	albumin (hur	aetna	O	3334.84	50.62	52.15	130.83	2501.13	
7	2/18/22	d5077cc8-85		75-2922687	J7040	cpt	normal salin	aetna	I	429.59	34.52	116.25	409	322.19	
8	2/18/22	d5077cc8-85		75-2922687	J7040	cpt	normal salin	aetna	O	394.14	21.88	230.35	230.35	295.61	
9	2/18/22	d5077cc8-85		75-2922687	P9035	cpt	platelet pher	aetna	O	962.47	499.61	N/A	499.61	721.85	
10	2/18/22	d5077cc8-85		75-2922687	S0020	cpt	injection, bu	aetna	O	262	11.57	90.78	249	196.5	
11	2/18/22	d5077cc8-85		75-2922687	P9045	cpt	albumin (hur	aetna	O	2428.86	50.12	52.18	53.16	1821.65	
12	2/18/22	d5077cc8-85		75-2922687	U0005	cpt	infec agen d	aetna	O	25.39	4.64	25	25	19.04	
13	2/18/22	d5077cc8-85		75-2922687	U0003	cpt	cov-19 amp	aetna	O	102.32	23.38	49.97	85.75	76.74	
14	2/18/22	d5077cc8-85		75-2922687	J7050	cpt	normal salin	aetna	O	285.46	36.52	142.51	237.89	214.1	
15	2/18/22	d5077cc8-85		75-2922687	J7050	cpt	normal salin	aetna	I	572.09	26.34	515.68	515.68	429.07	
16	2/18/22	d5077cc8-85		75-2922687	J9181	cpt	etoposide inj	aetna	I	3630	1125.3	1125.3	1125.3	2722.5	
17	2/18/22	d5077cc8-85		75-2922687	J7512	cpt	prednisone i	aetna	O	3	0.66	N/A	0.66	2.25	
18	2/18/22	d5077cc8-85		75-2922687	Q5106	cpt	inj retacrit n	aetna	I	424.5	8.3	269.7	269.7	318.38	
19	2/18/22	d5077cc8-85		75-2922687	U0002	cpt	covid-19 lab	aetna	O	91.89	5.62	63.42	63.42	68.92	
20	2/18/22	d5077cc8-85		75-2922687	J7060	cpt	5% dextrose,	aetna	I	768.69	27.4	486.16	486.16	576.52	
21	2/18/22	d5077cc8-85		75-2922687	M0243	cpt	casirivi and i	aetna	O	2173.56		N/A		1630.17	
22	2/18/22	d5077cc8-85		75-2922687	J9045	cpt	carboplatin i	aetna	I	15000	4650	4650	4650	11250	
23	2/18/22	d5077cc8-85		75-2922687	Q3014	cpt	telehealth fa	aetna	O	89	36.69	N/A	51.47	66.75	
24	2/18/22	d5077cc8-85		75-2922687	J7030	cpt	normal salin	aetna	O	527.12	32.2	368.01	466.88	395.34	
25	2/18/22	d5077cc8-85		75-2922687	J7030	cpt	normal salin	aetna	I	674.21	42.19	244.68	669.2	505.66	
26	2/18/22	d5077cc8-85		75-2922687	Q9957	cpt	inj perflutrer	aetna	O	714	215.27	N/A	215.27	535.5	
27	2/18/22	d5077cc8-85		75-2922687	J7120	cpt	ringers lacta	aetna	O	694.48	79.11	N/A	256.18	520.86	
28	2/18/22	d5077cc8-85		75-2922687	Q9963	cpt	hocm 350-39	aetna	O	3	0.32	N/A	0.32	2.25	
29	2/18/22	d5077cc8-85		75-2922687	J7042	cpt	5% dextrose,	aetna	I	703.5	33.73	N/A	477.02	527.63	
30	2/18/22	d5077cc8-85		75-2922687	Q4126	cpt	memoderm/	aetna	O	296	296	N/A	296	222	
31	2/18/22	d5077cc8-85		75-2922687	84484	cpt	Troponin (pr	state farm	I	650.68		N/A		488.01	
32	2/18/22	d5077cc8-85		75-2922687	P9016	cpt	rbc leukocyte	aetna	O	504.54	103.15	187.42	339.14	378.41	
33	2/18/22	d5077cc8-85		75-2922687	J7121	cpt	5% dextrose	aetna	I	2172.5	458.2	458.2	844.89	1629.38	
34	2/18/22	d5077cc8-85		75-2922687	S0077	cpt	injection, clir	aetna	O	553.7	56.02	262.73	262.73	415.28	
35	2/18/22	d5077cc8-85		75-2922687	Q9967	cpt	locm 300-39	aetna	O	119.55	0.12	N/A	44.13	89.66	
36	2/18/22	d5077cc8-85		75-2922687	J7070	cpt	d5w infusion	aetna	I	636.25	34.19	N/A	531	477.19	
37	2/18/22	d5077cc8-85		75-2922687	Q0138	cpt	ferumoxytol,	aetna	I	2554.7	1479	1479	1479	1916.03	
38	2/18/22	d5077cc8-85		75-2922687	P9017	cpt	plasma 1 do	aetna	O	227		N/A		170.25	
39	2/18/22	d5077cc8-85		75-2922687	37226	cpt	Insertion of s	aetna	O	135458.03	9723.21	12165.2	17606.66	101593.52	
40	2/18/22	d5077cc8-85		75-2922687	44378	cpt	Control of bl	aetna	O	22131.44	1425.1	1425.1	2952.95	16598.58	
41	2/18/22	d5447cc8-85		75-2922687	37227	cpt	Removal of p	aetna	O	151863.45	12732.07	15447.24	59274.99	113897.59	
42	2/18/22	d5077cc8-85		75-2922687	37229	cpt	Removal of p	aetna	O	126232.12	7795.8	15206.91	71178	94674.09	
43	2/18/22	d5077cc8-85		75-2922687	802	revCode	Inpatient Rei	aetna	I	1103		N/A		827.25	
44	2/18/22	d5077cc8-85		75-2922687	37231	cpt	Removal of p	aetna	O	164614.71	14945.28	15391.24	15391.24	123461.03	

Part of our created Excel file:

	Aetna HMO				Aetna PPO				Cigna HMO			
Hospital Name	DRG 807	CPT 45380	CPT 29827	CPT 70553	DRG 807	CPT 45380	CPT 29827	CPT 70553	DRG 807	CPT 45380	CPT 29827	CPT 70553
	NA	NA	NA	1904.5	NA	NA	NA	1904.5	8575.99	NA	NA	NA
	NA	NA	NA	NA	NA	NA	NA	NA	NA	NA	NA	NA
	NA				NA				NA			
				NA				NA				NA
		NA	NA	NA		NA	NA	NA	NA	1286	4975	NA
			NA	NA			NA	NA			NA	
	11930.23	1040.36	NA	1222	11794	3047	NA	2350	10001.8	1410	NA	1583
	NA	NA	NA	3075.3	NA	NA	NA	4824	3930	NA	NA	3497.4
				NA				NA	NA	NA	NA	NA
	NA	NA	NA	NA	NA	NA	NA	NA	NA	NA	NA	NA
	NA	NA	NA	NA	NA	NA	NA	NA	NA	NA	NA	NA
	NA	NA	NA	NA	NA	NA	NA	NA	NA	NA	NA	NA
	NA	NA	NA	2285.37	NA	NA	NA	NA	NA	NA	NA	196
	NA	NA	NA	NA	NA	NA	NA	NA	NA	NA	NA	3497.4
	NA	NA	NA	NA	NA	NA	NA	NA	NA	NA	NA	3498
	9498.54	5102.72	2585.47	2804.36								
									6649.49	1522.55	9198.76	540.5
	4546	5175.97	NA	3113.44	4546	5175.97	NA	3113.44	5734	5670.85	NA	708
	NA	NA	NA	NA	NA	NA	NA	NA	NA	NA	NA	NA
	NA	NA	27366.55	2431.78	NA	NA	27366.55	2431.78	NA	NA	19561.65	1738.24
	9369	1800	2881	5072	9369	1800	2881	5072	9290	NA	NA	NA
	NA	NA	NA	3075.3	NA	NA	NA	4824	NA	NA	NA	3497.4
	9468	NA	NA	NA	9468	NA	NA	NA	8863	NA	NA	NA
	9411	NA	NA	NA	9411	NA	NA	NA	NA	NA	NA	NA
	7635	NA	NA	NA	7635	NA	NA	NA	8863	NA	NA	NA
	10168	NA	NA	NA	10168	NA	NA	NA	8863	NA	NA	NA
	NA	NA	NA		NA	NA	NA		NA	NA	NA	
	NA	NA	NA	NA	7885.53	3132.8	8200	1400	4048	1286	4975	NA
	6714.42	NA	NA	3075.3	6714.42	NA	NA	4824	6714.42	NA	NA	3497.4
	NA				NA				NA			
	NA	NA	NA	NA	NA	NA	NA	NA	NA	NA	NA	NA
	NA	NA	NA	3075.3	NA	NA	NA	4824	NA	NA	NA	3135.6

	Cigna PPO				FL Blue HMO				FL Blue PPO			
Hospital Name	DRG 807	CPT 45380	CPT 29827	CPT 70553	DRG 807	CPT 45380	CPT 29827	CPT 70553	DRG 807	CPT 45380	CPT 29827	CPT 70553
	8575.99	NA	NA	77.86	7043.77	NA	NA	77.86	9051.69	NA	NA	NA
	NA	NA	NA	NA	NA	NA	NA	NA	NA	NA	NA	NA
	NA				NA				NA			
				NA	4516.63	1006.43	6080.49	NA	6000	1839.41	4390	NA
	NA	1286	4975	NA	NA	NA	NA	NA	NA	3484	7918	NA
			NA			NA	NA	NA		NA	NA	NA
	12448.88	1453	NA	1637	3161	3047	NA	2350	8614.53	3411	NA	2889
	NA	NA	NA	4824	NA	NA	NA	3618	3249	NA	NA	3907.44
	NA	NA	NA	NA	NA	NA	NA	NA	9119.94	NA	NA	NA
	NA	NA	NA	NA	NA	NA	NA	NA	NA	NA	NA	NA
	NA	NA	NA	NA	NA	NA	NA	NA	NA	NA	NA	NA
	NA	NA	NA	NA	NA	NA	NA	NA	NA	NA	NA	NA
	NA	NA	NA	196	NA	NA	NA	280	NA	NA	NA	280
	NA	NA	NA	4824	NA	NA	NA	3618	NA	NA	NA	2907.44
	NA	NA	NA	4825	NA	NA	NA	3619	NA	NA	NA	NA
					NA	NA	NA	NA	NA	NA	NA	NA
					NA	NA	NA	NA	NA	NA	NA	NA
					NA	NA	NA	NA	13007.28	5658.69	2867.18	2146.41
	6649.49	1522.55	9198.76	540.5	NA	NA	NA	NA	NA	NA	NA	NA
	5734	5670.85	NA	708	9247.4	1013.85	NA	359.91	9338.06	1023.79	NA	363.44
	NA	NA	NA	NA	7107.44	NA	NA	NA	7107.44	NA	NA	NA
	NA	NA	19561.65	1738.24	NA	NA	21833.96	1940.16	NA	NA	21833.96	1940.16
	NA	NA	NA	NA	NA	NA	NA	NA	8733	2746	12972	1316
	NA	NA	NA	4824	NA	NA	NA	3618	NA	NA	NA	NA
	NA	4230	NA	5753	NA	NA	NA	NA	11778	NA	NA	NA
	NA	4230	NA	5753	NA	NA	NA	NA	11582	NA	NA	NA
	NA	4230	NA	5753	NA	NA	NA	NA	9886	NA	NA	NA
	NA	4230	NA	5753	NA	NA	NA	NA	12320	NA	NA	NA
	NA	NA	NA		NA	NA	NA	NA	9529.95	3573	8166	NA
	4048	1286	4975	NA	6550.76	NA	NA	NA	8075.3	NA	NA	NA
	6714.42	NA	NA	4824	6714.42	NA	NA	3618	3249	NA	NA	3907.44
	NA				NA	NA	NA	NA	NA	NA	NA	NA
	NA	NA	NA	NA	NA	NA	NA	NA	NA	NA	NA	NA
	NA	NA	NA	4824	NA	NA	NA	3618	NA	NA	NA	NA

	United Healthcare HMO				United Healthcare PPO				
Hospital Name	DRG 807	CPT 45380	CPT 29827	CPT 70553	DRG 807	CPT 45380	CPT 29827	CPT 70553	Region
	NA	NA	NA	1576.93	NA	NA	NA	1576.93	SOUTH
	4775.27	NA	NA	NA	4775.27	NA	NA	NA	WEST COAST
	NA	474.38	1267.61	395.03	NA	474.38	1267.61	395.03	WEST COAST
				NA				NA	CENTRAL
									SOUTH
	8751.02	4206	12983	NA	7560	4206	12983	NA	NORTHWEST
		NA	NA	NA		NA	NA	NA	NORTHEAST
			NA				NA		WEST COAST
	NA	NA	NA	NA	NA	NA	NA	NA	SOUTH
	7410.03	NA	NA	NA	NA	NA	NA	NA	NORTHWEST
	NA	NA	NA	NA	NA	NA	NA	NA	NORTHEAST
	NA	NA	NA	NA	NA	NA	NA	NA	NORTHEAST
	NA	NA	NA	NA	NA	NA	NA	NA	NORTHEAST
	NA	NA	NA	NA	NA	NA	NA	NA	SOUTH
	NA	NA	NA	NA	NA	NA	NA	NA	SOUTH
	NA	NA	NA	NA	NA	NA	NA	NA	WEST COAST
									CENTRAL
									CENTRAL
									CENTRAL
									CENTRAL
	NA	NA	NA	NA	NA	NA	NA	NA	SOUTH
	NA	NA	NA	NA	NA	NA	NA	NA	SOUTH
	NA	NA	20549.61	1826.03	NA	NA	20549.61	1826.03	WEST COAST
									WEST COAST
	NA	NA	NA	NA	NA	NA	NA	NA	SOUTH
	NA	NA	NA	NA	NA	4794	NA	6520	SOUTH
	NA	NA	NA	NA	NA	4794	NA	6520	SOUTH
	NA	NA	NA	NA	NA	4794	NA	6520	SOUTH
	NA	NA	NA	NA	NA	4794	NA	6520	SOUTH
	3614.64	967.83	5765.17	368.01	3614.64	967.83	5765.17	368.01	WEST COAST
	9665.86	3132.8	9818	NA	NA	NA	NA	NA	NORTHWEST
	NA	NA	NA	NA	NA	NA	NA	NA	SOUTH
	NA				NA				NORTHEAST
	NA	NA	NA	NA	NA	NA	NA	NA	CENTRAL
	NA	NA	NA	NA	NA	NA	NA	NA	SOUTH
