# Supplementary material for: Usability of Health Care Price Transparency Data in the United States: Mixed Methods Study
Source: J Med Internet Res. 2024 Mar 29;26:e50629. doi: 10.2196/50629 (PMC11015359; doi:10.2196/50629)

Multimedia Appendix 2: Survey questions and experiment scenarios

Survey questions:

1. Have you heard about the price transparency rule in healthcare?
   - o Yes
   - o No

2. Do you know it is your right to know the cost of healthcare services before you receive them?
   - o Yes
   - o No

3. Do you know that you can find the cost of a service on the hospital's website?
   - o Yes
   - o No

4. Have you ever forgone needed healthcare services due to cost/price uncertainty?
   - o Yes
   - o No

5. Have you ever used a service and discovered that the hospital where you received the service was more expensive than other healthcare centers?
   - o Yes
   - o No

6. Have you ever searched for a service price prior to going to the hospital?
   - o Yes
   - o No

7. Which one of the following methods have you used to get access to the price of a service? (check all that apply)
   - ❑ Advertisement
   - ❑ Hospital' website or Tools
   - ❑ Asking friends or relatives or other patients
   - ❑ Contacting hospital
   - ❑ Contacting insurance company
   - ❑ Other:

8. In thinking about your answer to the previous question, are any of these methods easily available and provide you updated, real-time prices?
   - o Yes
   - o No

9. If you utilized a hospital website to obtain healthcare service pricing, did you find the pricing information easy to use and comprehensive?
   - o Yes

- No
- N/A

10. Do you know how to access reliable sources to obtain estimated healthcare service prices?
    - Yes
    - No

11. Do you know you can find service prices on hospitals' websites?
    - Yes
    - No

12. Have you ever received a medical bill that you may not expect to charge for?
    - Yes
    - No

Experiment scenarios and questions:

Please answer three following questions based on the given scenario.
Scenario #1: Imagine your friend was asked by his/ her doctor to take an MRI of his/her brain, and he/she asks you to get an appointment from a hospital for next month.
You would want to search for the service name "MRI scan of brain before and after contrast")
1. Please search for the required service through <u>this file</u> / <u>this link</u> and fill the following blanks about your choice.
Hospital Name:
Service price:
Insurance Company Name:
Insurance Plan:

2. Was it simple to choose the appropriate hospital with a reasonable price?
    - Yes
    - No

3. Did you choose the minimum cost? If "No" please write your reason.
    - Yes
    - No, why?
    -

Please answer three following questions based on the given scenario.
Scenario #2: Imagine your friend is going to have a baby within the next two months, and your friend asks for your help to find a hospital.
You would want to search for the service name "vaginal delivery without sterilization"
1. Please search for the required service through <u>this file</u> / <u>this link</u> and fill the following blanks about your choice.
Hospital Name:
Service price:
Insurance Company Name:

Insurance Plan:
2. Was it simple to choose the appropriate hospital with a reasonable price?
- o Yes
- o No

3. Did you choose the minimum cost? If "No" please write your reason.
- o Yes
- o No, why?

Please answer three following questions based on the given scenario.
Scenario #3: Imagine your friend has a pain in his/ her shoulder, and he/ she was asked to have surgery next month. Your friend asks for your help to find a hospital for his/ her surgery.
You would want to search for the service name "Arthroscopic cuff repair (shoulder arthroscopy)".
1. Please search for the required service through this file / this link and fill the following blanks about your choice.
Hospital Name:
Service price:
Insurance Company Name:
Insurance Plan:
2. Was it simple to choose the appropriate hospital with a reasonable price?
- o Yes
- o No

3. Did you choose the minimum cost? If "No" please write your reason.
- o Yes
- o No, why?

4. Imagine the service quality is the same among all hospitals; please rank the following items based on their importance that affect your hospital selection. (You may write "1" for the highest priority and "4" for the lowest.)
- — Service price
- — Hospital distance
- — Insurance coverage
- — Hospital familiarity/ Hospital Brand

5. Do you know how to find mentioned service price from the hospitals' websites or price transparency tools?
- o Yes
- o No

6. How much prior knowledge do you think you need before searching for specific services?
- o No knowledge required
- o A little
- o Moderate knowledge
- o You need to be an expert

7. Have you ever used price comparison tools to find a specific service price?

- Yes
- No

8. Have you ever postponed receiving healthcare services because of the price?

- Yes
- No

9. Does price transparency help you in deciding where to obtain a healthcare service?

- Yes
- No

10. Which factor of this file do you think is more helpful? (check all that apply)

- ❑ Gross charge
- ❑ Discounted cash price
- ❑ Minimum negotiated price
- ❑ Maximum negotiated price
- ❑ Insured price

11. Please mention any other factors you believe should be included in these files in addition to the pricing.

12. How likely are you to recommend this system to a friend?

- Not likely at all
- Neutral
- Extremely likely

13. Do you agree that price transparency in healthcare reduces your potential out-of-pocket expenses?

- No, I do not agree.
- Yes, I agree.

14. Overall, do you prefer to utilize the files or price transparency tools to identify future service prices now that you have a better grasp of this regulation?

- Yes
- No

Pilot Demographics Charts

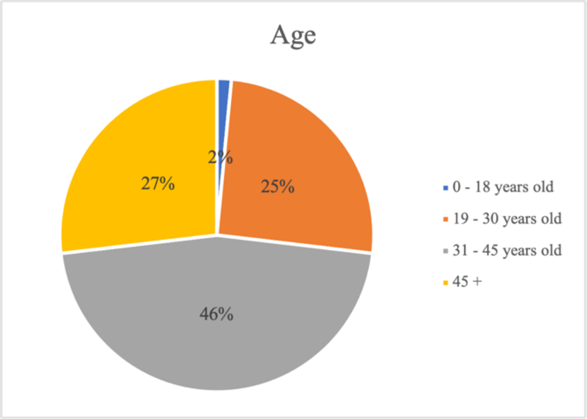

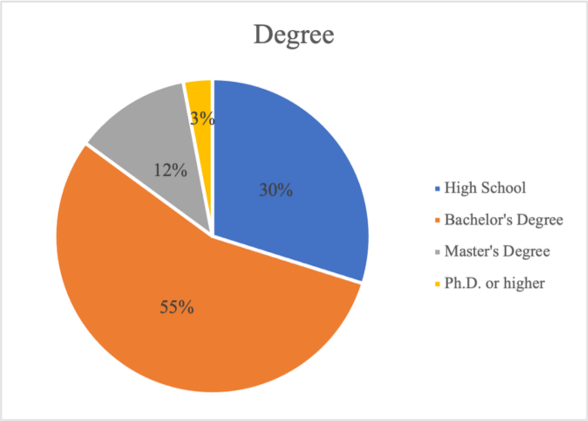

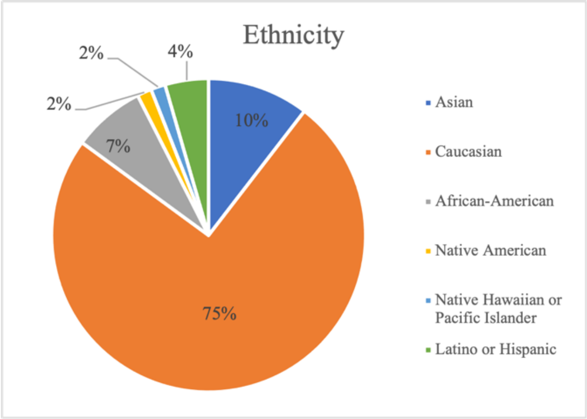

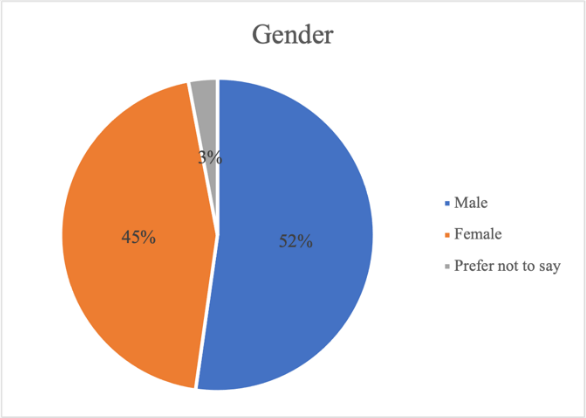

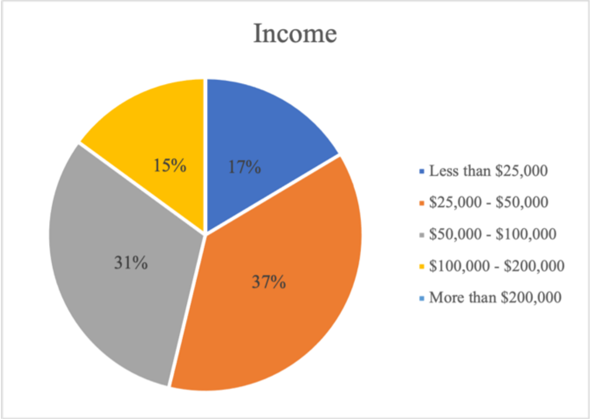

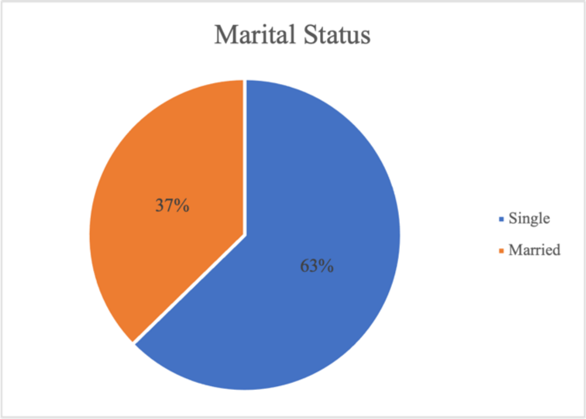

Supplement: Multimedia Appendix 2 [file jmir_v26i1e50629_app2.pdf]
