# Supplementary figures and images for: Usability of Health Care Price Transparency Data in the United States: Mixed Methods Study
Source: J Med Internet Res. 2024 Mar 29;26:e50629. doi: 10.2196/50629 (PMC11015359; doi:10.2196/50629)

### Multimedia Appendix 3: Participants' responses chart regarding PT awareness

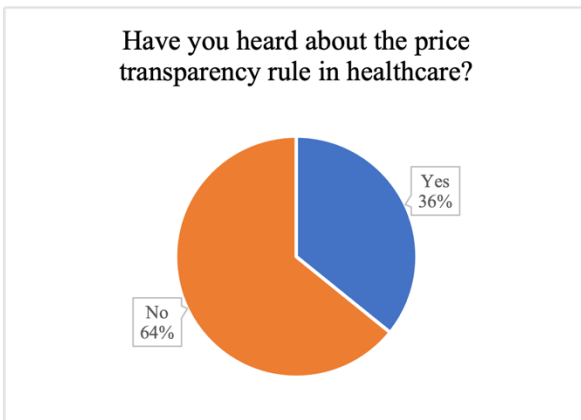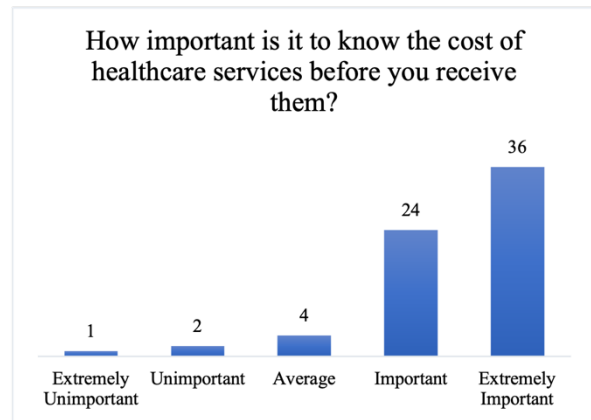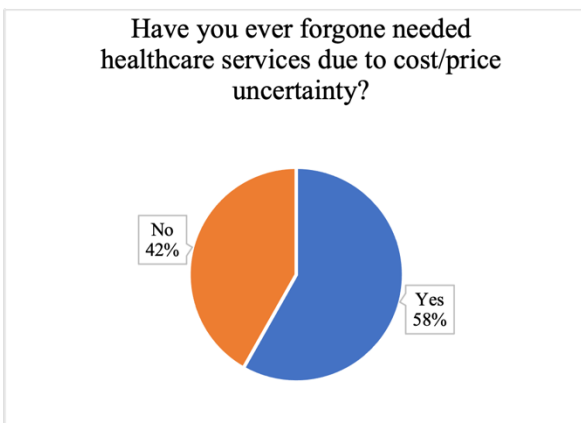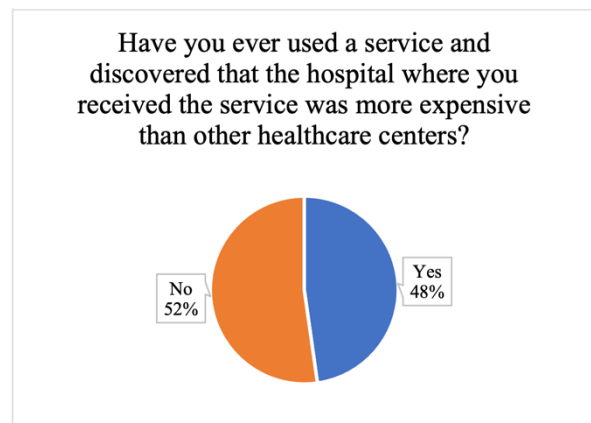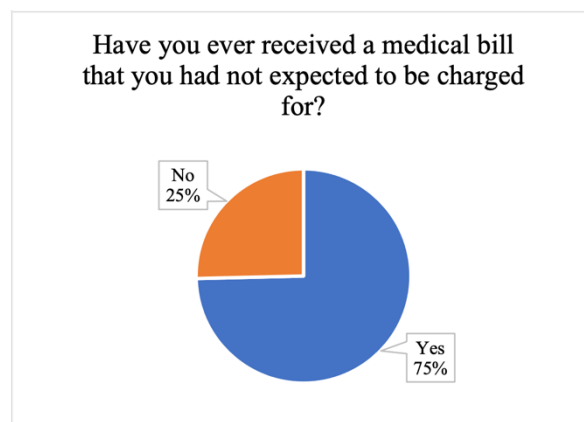

Supplement: Multimedia Appendix 3 [file jmir_v26i1e50629_app3.pdf]
