# Supplementary material for: Usability of Health Care Price Transparency Data in the United States: Mixed Methods Study
Source: J Med Internet Res. 2024 Mar 29;26:e50629. doi: 10.2196/50629 (PMC11015359; doi:10.2196/50629)
Supplement: Multimedia Appendix 4 [file jmir_v26i1e50629_app4.pdf]

Multimedia Appendix 4: T-test analysis regarding human usability of PT information based on participants' responses

Scenarios

- H_0: $\mu_{control\ Gp} = \mu_{treatment\ Gp}$
- H_1: $\mu_{control\ Gp} \neq \mu_{treatment\ Gp}$

t-Test: Two-Sample Assuming Equal Variances		
	Variable 1	Variable 2
Mean	2.75925926	1.230769231
Variance	0.3371768	1.692307692
Observations	54	13
Pooled Variance	0.58735481	
Hypothesized Mean Difference	0	
df	65	
t Stat	6.45570237	
P(T<=t) one-tail	7.8484E-09	
t Critical one-tail	1.66863598	
P(T<=t) two-tail	1.5697E-08	
t Critical two-tail	1.99713791	

How much prior knowledge do you think you need to compare the price of a specific service with the tools you have used in this survey?

- H_0: $\mu_{control\ Gp} = \mu_{treatment\ Gp}$
- H_1: $\mu_{control\ Gp} \neq \mu_{treatment\ Gp}$

t-Test: Two-Sample Assuming Equal Variances		
	Variable 1	Variable 2
Mean	2.51851852	3.230769231
Variance	0.82040531	0.692307692
Observations	54	13
Pooled Variance	0.79675652	
Hypothesized Mean Difference	0	
df	65	
t Stat	-2.5828622	
P(T<=t) one-tail	0.00602898	
t Critical one-tail	1.66863598	
P(T<=t) two-tail	0.01205796	
t Critical two-tail	1.99713791	

How useful is price transparency in deciding where to obtain a healthcare service?

- H_0: $\mu_{control\ Gp} = \mu_{treatment\ Gp}$
- H_1: $\mu_{control\ Gp} \neq \mu_{treatment\ Gp}$

t-Test: Two-Sample Assuming Equal Variances		
	Variable 1	Variable 2
Mean	4.35185185	3.692307692
Variance	0.68518519	1.064102564
Observations	54	13
Pooled Variance	0.75513916	
Hypothesized Mean Difference	0	
df	65	
t Stat	2.45675323	
P(T<=t) one-tail	0.00834948	
t Critical one-tail	1.66863598	
P(T<=t) two-tail	0.01669896	
t Critical two-tail	1.99713791	

How likely are you to recommend the price transparency tools you have used to a friend for their medical requirement?

- H_0: $\mu_{control\ Gp} = \mu_{treatment\ Gp}$
- H_1: $\mu_{control\ Gp} \neq \mu_{treatment\ Gp}$

t-Test: Two-Sample Assuming Equal Variances		
	Variable 1	Variable 2
Mean	4.24074074	3.692307692
Variance	0.94095038	1.064102564
Observations	54	13
Pooled Variance	0.96368617	
Hypothesized Mean Difference	0	
df	65	
t Stat	1.8083686	
P(T<=t) one-tail	0.03758754	
t Critical one-tail	1.66863598	
P(T<=t) two-tail	0.07517507	
t Critical two-tail	1.99713791	

How likely is it that price transparency in healthcare can reduce your potential out-of-pocket expenses?

- H_0: $\mu_{control\ Gp} = \mu_{treatment\ Gp}$
- H_1: $\mu_{control\ Gp} \neq \mu_{treatment\ Gp}$

t-Test: Two-Sample Assuming Equal Variances		
	Variable 1	Variable 2
Mean	4.38888889	3.538461538
Variance	0.84591195	1.602564103
Observations	54	13
Pooled Variance	0.98560158	
Hypothesized Mean Difference	0	
df	65	
t Stat	2.77279447	
P(T<=t) one-tail	0.00362195	
t Critical one-tail	1.66863598	
P(T<=t) two-tail	0.00724389	
t Critical two-tail	1.99713791	

Overall, how likely will you use the files or the links provided to identify future service prices now that you have a better grasp of this regulation?

- H_0: $\mu_{control\ Gp} = \mu_{treatment\ Gp}$
- H_1: $\mu_{control\ Gp} \neq \mu_{treatment\ Gp}$

t-Test: Two-Sample Assuming Equal Variances		
	Variable 1	Variable 2
Mean	3.96296296	3
Variance	1.28162124	1.5
Observations	54	13
Pooled Variance	1.32193732	
Hypothesized Mean Difference	0	
df	65	
t Stat	2.71103614	
P(T<=t) one-tail	0.00428527	
t Critical one-tail	1.66863598	
P(T<=t) two-tail	0.00857054	
t Critical two-tail	1.99713791	
